# Antitumour Effects of β-Blockers: A Narrative Review of the Literature

**DOI:** 10.3390/cimb48090865

**Published:** 2026-08-26

**Authors:** Dimitra Melissaridou, Olga D. Savvidou, Ioanna Lianou, Angelos Kaspiris, Penelope Korkolopoulou, Evangelia Papadimitriou, Panayiotis J. Papagelopoulos

**Affiliations:** 1 First Department of Orthopaedic Surgery, School of Medicine, National and Kapodistrian University of Athens, “ATTIKON” University Hospital, Rimini 1, 12462 Athens, Greece; dimitramel@med.uoa.gr (D.M.); olgasavvid@med.uoa.gr (O.D.S.); pjportho@med.uoa.gr (P.J.P.); 2 Orthopaedics and Traumatology, “Agios Andreas” General Hospital of Patras-National Health Service (NHS), 262224 Patras, Greece; jlianou@med.uoa.gr; 3 Third Department of Orthopaedic Surgery, School of Medicine, National and Kapodistrian University of Athens, “KAT” General Hospital, Nikis 2, 14561 Athens, Greece; 4 First Department of Pathology, School of Medicine, National and Kapodistrian University of Athens, 75 Mikras Asias Street, Goudi, 11527 Athens, Greece; pkorkol@med.uoa.gr; 5 Laboratory of Molecular Pharmacology, Department of Pharmacy, School of Health Sciences, University of Patras, 26504 Patras, Greece; epapad@upatras.gr

**Keywords:** β-blocker, adrenergic receptors, tumour, cancer, metastasis

## Abstract

Beta (β)-adrenergic receptors are Gs-protein-coupled receptors with many physiological actions but are also expressed in various types of cancers. Preclinical *in vitro* and *in vivo* research studies demonstrated that the antagonism of β-adrenergic receptor signalling by β-blockers inhibits multiple cellular processes involved in cancer progression, including tumour cell proliferation, extracellular matrix invasion, matrix metalloproteinase (MMP) activation, expression of inflammatory or chemotactic cytokines, and angiogenesis, thus regulating the growth of tumours and reducing the development of metastasis in a dose-dependent manner. Furthermore, several experimental studies reported that in patients treated with the combination of neoadjuvant chemotherapy, targeted therapies, and β-blockers, the risk of cancer recurrence and metastasis development was reduced and associated with a decreased rate of mortality. This review aims to compile and critically evaluate preclinical and clinical research of the association between β-blocker administration and tumour growth across various tumour types.

## 1. Introduction

Despite the progress in the early diagnosis of the majority of tumours and the progressively more effective treatment options, cancer is estimated to be one of the most common causes of death [1]. Efforts to develop new drugs are intense, but the process is complex and time-consuming, and only a few advance to later phases of clinical trials [2]. Another approach is to investigate the potential of medicines approved for other indications, such as metformin, disulfiram, aspirin, thalidomide, raloxifene, and β-blockers, for repurposing in cancer treatment [3,4,5]. This review focuses on the existing literature related to the potential anticancer activities of beta (β)-blockers.

The β-adrenergic system seems to influence cancer progression in every step of tumour development. Adrenergic receptors are expressed in cancer and immune cells and, upon activation, e.g., by chronic stress, influence proliferation, mobility, in signalling pathways involved in cancer invasion, metastasis, angiogenesis, and lymphangiogenesis [6,7,8]. The activation of β_2_-adrenergic signalling may also suppress natural killer (NK) cell production, affect T-cell proliferation and display remarkable negative effect on CD8^+^ T-cell function, while it raises the expression of programmed death-1 (PD-1), ultimately resulting in poorer control of tumour growth [9].

β-Blockers are classified according to their selectivity for β-adrenergic receptors, intrinsic sympathomimetic activity, lipid solubility, and additional vasodilatory properties. Based on receptor selectivity, they are divided into three generations. First-generation agents, such as propranolol and nadolol, are non-selective and antagonise both β1- and β2-adrenergic receptors. Second-generation β-blockers, including atenolol, metoprolol, and bisoprolol, preferentially inhibit β1 receptors, which are expressed predominantly in cardiac tissue. Third-generation β-blockers, such as carvedilol and nebivolol, possess additional pharmacological properties, including α1-adrenergic receptor antagonism, nitric oxide-mediated vasodilation, antioxidant activity, and other pleiotropic effects that may further influence tumour biology [6,10,11,12]. Receptor selectivity appears to be an important determinant of the anticancer effects of β-blockers. Experimental evidence indicates that β2-adrenergic signalling plays a central role in promoting tumour cell proliferation, angiogenesis, epithelial–mesenchymal transition, invasion, metastasis, and immune evasion through activation of downstream signalling pathways, including cAMP/protein kinase A (PKA), mitogen-activated protein kinase/extracellular signal-regulated kinase (MAPK/ERK), phosphatidylinositol 3-kinase/protein kinase B (PI3K/AKT), and nuclear factor kappa B (NFκB) [10,13]. Consequently, non-selective β-blockers, particularly propranolol, have demonstrated more consistent antitumour activity in preclinical studies and have shown encouraging results in several retrospective clinical studies, supporting their potential repositioning as adjuvant anticancer therapies [14,15,16]. Understanding these pharmacological differences is therefore essential for interpreting the heterogeneous findings reported across experimental and clinical studies and for identifying the β-blocker subclasses with the greatest potential for drug repositioning in oncology.

This review aims to summarise, evaluate, and discuss existing evidence on the role of β-blockers as adjuvant therapies in cancer. It examines findings from various approaches, including *in vitro* and *in vivo* research, as well as preclinical and clinical studies, across different tumour types.

## 2. Materials and Methods

This narrative review was conducted to provide a comprehensive overview of the available evidence regarding the potential antitumour effects of β-blockers in oncology. A structured literature search was performed in PubMed and Web of Science on 15 April 2026 to identify relevant publications. The search strategy combined the following keywords and Medical Subject Heading (MeSH) terms where appropriate: “β-blockers”, “beta-blockers”, “cancer”, “tumour”, “tumor”, “metastasis”, “angiogenesis”, “survival”, “chemotherapy”, “immunotherapy”, and “therapy”. Reference lists of relevant articles were also screened to identify additional eligible publications. Duplicate records were removed using Zotero (version 6.0.10; Digital Scholar).

Eligible articles included original experimental studies (*in vitro* and *in vivo*), observational clinical studies, clinical trials, systematic reviews, and meta-analyses evaluating the effects of β-blockers on tumour biology, cancer progression, treatment response, or patient outcomes. Only full-text articles published in English were considered. Case reports, case series, editorials, conference abstracts, book chapters, letters to the editor, and publications with insufficient methodological or outcome data were excluded. No restrictions were applied regarding the year of publication to provide a comprehensive overview of the available evidence.

Relevant information from the selected publications was extracted by the authors and organised according to study design, tumour type, β-blocker evaluated, principal molecular mechanisms, therapeutic combinations, and reported clinical or experimental outcomes. The evidence was subsequently synthesised narratively to summarise current knowledge regarding the potential role of β-blockers as adjuvant anticancer therapies, identify areas of agreement and controversy, and highlight directions for future research.

Artificial intelligence was not used to support preparation of the manuscript.

## 3. Results

### 3.1. Preclinical Studies

#### 3.1.1. Preclinical *In Vitro* and *In Vivo* Studies on Tumour Growth and Apoptosis

β-Blockers can act directly on tumour cells or affect the tumour microenvironment. Several preclinical studies show evidence of a direct antitumour role of β-blockers [1,11,17,18,19,20,21,22,23,24,25,26,27,28,29,30,31,32,33,34,35,36,37,38] (Table 1). According to Liu and Jan, carvedilol, a non-specific β-blocker, increases the intracellular concentration of Ca^2+^ in osteosarcoma cells by increasing the influx of extracellular and the release from intracellular resources, leading to inhibition of cell proliferation and cytotoxicity [32]. The anti-proliferative role of propranolol, a non-selective β-blocker, in infantile haemangioma depends on its action on haemangioma endothelial cells and tumour stem-cells via reducing cAMP levels and increasing extracellular signal-regulated kinase (ERK)1/2, resulting in stem cell apoptosis and rescue of the development of abnormal endothelial vessels [35]. Similarly, propranolol, but not the cardioselective metoprolol, inhibits proliferation and enhances melanoma cell death *in vitro*, while it has a minor effect on normal skin or primary melanocytes [37]. In addition to β1 and β2 subtypes, β3-adrenergic receptors also play a significant role in melanoma malignancy by regulating motility and stem-cell traits of melanoma cells [21,39]. In melanoma patients’ biopsies, β2-adrenergic receptors are expressed on melanoma cells, and wide-spectrum β-blockers inhibit melanoma cell proliferation [21].

In human lung adenocarcinoma A549 cells, propranolol inhibits cell viability through both caspase-dependent and independent apoptosis pathways, while it has a significantly lesser effect on BEAS2B nontumour lung epithelial cells [29]. On the other hand, carvedilol had modest-to-no inhibitory effect on the growth of human lung cancer A549 tumours *in vivo* and *in vitro* [32].

Carvedilol, but not atenolol, has been shown to have preventive activity against skin cancer development through β2-adrenergic receptors expressed on JB6 P(+) cells, a skin cell model. The preventive effect was observed *in vivo* and was more potent upon topical application of the drug [32]. These data suggest that although carvedilol may not be an effective treatment for established tumours, it may have a preventive effect, as in the case of skin cancer.

#### 3.1.2. Preclinical Studies on the Effects of β-Blockers on the Tumour Microenvironment

The effect of β-blockers on the suppression of specific tumour types may also be related to their effect on the tumour microenvironment. For example, the increased recruitment of fibroblasts, mesenchymal stem cells, monocytes, and endothelial cells by conditioned medium from human melanoma A375 cells stimulated with norepinephrine was inhibited by β-blockers, with a more prominent effect observed with a selective β3-blocker compared with a selective β2-blocker or the non-selective propranolol [33].

Propranolol has also been shown to decrease the fraction of myeloid suppressor cells and increase the fraction of cytotoxic lymphoid cells that infiltrate the tumour and show antitumour action. An additional antitumour action of β-blockers appears to be the reduction in CD34^+^ cells involved in angiogenesis, as stromal cells, including fibroblasts, may impair melanoma growth and slow disease progression [37]. The inhibition of β2-adrenergic receptors by propranolol has also been shown to enhance the functionality of CD8^+^ T cells, enhancing antitumour immunity. Macrophages may increase β-adrenergic signalling in the tumour microenvironment, and propranolol was found to increase the infiltration of T-lymphocytes and their cytotoxic activity in tumours [40].

Moreover, several effector lymphocytes, such as CD8^+^, natural killer (NK) cells, and Tγδ cells, are activated by catecholamines via β2-adrenergic receptors in response to exercise [41,42]. These cells are effective in recognising and destroying malignant cells. Non-selective β-blockers tend to inhibit lymphocyte activation and mobilisation during exercise, whereas isoproterenol and epinephrine induce it. According to Smith et al., blockade of β1-adrenergic receptors combined with a phosphodiesterase-4 (PDE-4) inhibitor enhances the immobilisation of monocytes, CD8^+^, NK and Tγδ cells, even at low-intensity exercise [43]. Moreover, exercise was reported to suppress the growth of lymphomas in animal models (mice) via NK-cell activation and β2-adrenergic signalling. These findings contrast with the previous literature and emphasise the role of β1-blockade in the expansion of effector lymphocytes and the effect of non-selective β-blockers, which blunt the beneficial effect of exercise [43].

Interestingly, catecholamine-mediated β-adrenergic activation promotes the ex vivo expansion and mobilisation of Vγ9Vδ2 T cells, which demonstrate antitumour activity in humans and may serve as a potential immunotherapeutic approach for patients undergoing allogeneic hematopoietic cell transplantation as well as those with solid tumours [44].

#### 3.1.3. Role of β-Blockers Against Angiogenesis

β-Blockers can inhibit the proliferation, migration, and differentiation of endothelial cells into capillaries, a pathway normally driven by the growth factor vascular endothelial growth factor A (VEGFA) [45]. The beneficial effect of propranolol on haemangiomas of infancy is associated with its inhibitory effects on the abnormally activated endothelial cells [46]. However, the efficacy of propranolol in the treatment of infantile haemangiomas may be more complex than inhibition of angiogenesis alone, since its effects are not selective for endothelial cells [47]. Propranolol may also affect angiogenesis in other tumour types. For example, propranolol and, to a lesser degree, cardioselective β-blockers appear to suppress angiogenesis in melanoma [21,37].

#### 3.1.4. Synergistic Role of β-Blockers with Chemotherapy, Radiotherapy, and Immunotherapy

The concomitant use of β-blockers and chemotherapy appears to have synergistic antimitotic properties [40]. For example, a combination of propranolol with cisplatin had a synergistic effect on inhibiting osteosarcoma cell proliferation *in vitro*. In an *in vivo* mouse osteosarcoma model, it also resulted in enhanced cell-cycle arrest, disruption of mitogenic signalling cascades, apoptosis, and inhibition of cancer cell proliferation compared with each drug administered alone [25]. Propranolol also synergised with doxorubicin to inhibit the cell viability of liposarcoma and leiomyosarcoma cells. It also increased the response to docetaxel in angiosarcoma- and solitary fibrous tumour-patient-derived cells by reducing NFκB/COX-2 pathway activation [24]. Similarly, a selective β2-adrenergic receptor antagonist, ICI 118551, has been shown to enhance the cytotoxic and pro-apoptotic effects of gemcitabine in pancreatic cancer cells by inhibiting NFκB activation [33]. β-Blockers have also been linked to improved survival in patients with non-squamous cell carcinoma receiving radiotherapy, potentially due to their protective effects against radiotherapy-induced cardiac toxicity [48]. They may also act synergistically with immunotherapy, as experimental evidence suggests that inhibition of β2-adrenergic receptors enhances CD8^+^ T-cell function and improves tumour suppression [49].

#### 3.1.5. β-Blockers in Overcoming Chemoresistance

Failure of traditional chemotherapeutic agents is usually due to multidrug resistance (MDR) developed in cancer cells. Several β-adrenergic receptor antagonists, including carvedilol, atenolol, metoprolol, and propranolol, have been examined for reversing multidrug resistance (MDR) [50]. According to *in vitro* studies, carvedilol exerts an effect on MDR similar to that of verapamil; other β-blockers have no such activity, suggesting that this effect is independent of β-adrenergic receptor blockade and may instead be related to carvedilol’s antioxidant properties [27].

Propranolol reverses resistance mechanisms to chemotherapeutic agents used in various types of sarcomas by decreasing the activity of the MDR efflux pump P-glycoprotein, thus increasing the intracellular concentration and the antitumour activity of doxorubicin [24]. Results from *in vivo* and *in vitro* studies also reveal the contribution of propranolol to the anti-proliferative effect of trastuzumab on breast cancer cells and the re-sensitisation of resistant cells to trastuzumab [34].

More examples of all the above can be found in Table 1.

**Table 1:** Data from *in vivo* and *in vitro* preclinical models.

Study	Type of Study (Experimental Model)	Purpose of the Study	Β-Blocker Used	Tumour Studied	Results
Kakumoto et al., 2002 [27]	*In vitro* (Human cervical carcinoma cell lines)	To estimate the effect of β-blockers, including carvedilol, on MDR	Carvedilol, atenolol. propranolol, metoprolol	Cervical carcinoma	Only carvedilol inhibits MDR, an effect that seems to be independent of the β-adrenergic receptors
Liu and Jan, 2004 [32]	*In vitro* (MG63 human osteosarcoma cells)	To study the cytotoxic effect of carvedilol on MG63 osteosarcoma cells	Carvedilol	Osteosarcoma	Treatment with carvedilol at concentrations of 0.1–30 μΜ can inhibit cell proliferation by 13–40%, by inducing an increase in [Ca^2+^] concentration and cytotoxicity
Shan et al., 2011 [33]	*In vitro* (human pancreatic cancer cell lines)	To study the action of β2 adrenergic receptor blocker ICI 118551 on pancreatic cancer cells combined with gemcitabine	ICI 118551	Pancreatic cancer	ICI 118551 combined with gemcitabine induces apoptosis through inhibition of NFkB activation
Pasquier et al., 2013 [11]	*In vitro* (human neuroblastoma cell lines)	To evaluate whether β-blockers could enhance the efficiency of chemotherapy in the treatment of neuroblastoma	Carvedilol, labetalol propranolol, nebivolol, metoprolol, atenolol, butoxamine	Neuroblastoma	β-Βlockers exhibit strong anti-proliferative and *in vitro* anti-angiogenic activities against neuroblastoma
Lu et al., 2015 [51]	*In vivo* and *in vitro* (HER2-positive breast cancer cell lines and xenograft mouse models)	To study the molecular pathways related to trastuzumab resistance in HER2-overexpressing breast cancer cells	Propranolol	Breast cancer	Catecholamines antagonise the anti-proliferative role of trastuzumab, whereas propranolol re-sensitises resistant cells to trastuzumab and enhances its action
Munabi et al., 2015 [35]	*In vivo* and *in vitro* (haemangioma stem cells and murine haemangioma models)	To examine the mechanisms by which propranolol affects haemangioma stem-cell behaviour	Propranolol	Haemangioma	Propranolol has possible targets other than β-adrenergic receptors at high cytotoxic doses
Wilson et al., 2015 [36]	*In vitro* (human breast cancer cell lines)	To study how β-adrenergic receptors regulate Rap1 prenylation and thus promote breast cancer metastasis.	Propranolol	Breast cancer	β-Agonists promote metastasis by activating G protein-coupled receptors and decreasing Rap1B prenylation. Cell migration can be diminished by propranolol
Wrobel and Gal 2015 [37]	*In vitro* (human melanoma cell lines)	To evaluate the results from the use of propranolol on the proliferation and survival of human melanoma cell culture lines	Propranolol and metoprolol	Melanoma	Propranolol, but not metoprolol, may be a useful adjuvant for patients with high-risk melanoma
Chang et al., 2016 [38]	*In vitro* and *in vivo* (epidermal/skin cancer cell models and murine skin carcinogenesis model)	To study whether carvedilol, through activation of EGFR, can promote cancer	Carvedilol	Skin cancer	In non-toxic concentrations, carvedilol inhibits EGF-induced malignant transformation, suggesting its chemopreventive activity against skin cancer
Montoya et al., 2017 [17]	*In vitro* (human breast cancer cell lines)	To examine the anti-proliferative role of β-blockers on breast cancer cells	Propranolol	Breast cancer	Propranolol is more effective at reducing breast cancer cell viability than selective β-blockers
Rico et al., 2017 [18]	*In vitro* (triple-negative breast cancer cell lines and mouse models)	To study the combined role of metformin and propranolol on metastasis, survival, and tumour growth in triple-negative breast cancer	Propranolol and metformin	Triple-negative breast cancer	The combination of drugs is more effective in blocking proliferation and delaying tumour growth
Bustamante et al., 2019 [19]	*In vitro* (primary and metastatic uveal melanoma cell cultures)	To evaluate the effect of propranolol on primary and metastatic uveal melanoma	Propranolol	Uveal, cutaneous melanoma	Anti-proliferative, anti-migratory, and pro-apoptotic role of propranolol
Duckett et al., 2020 [20]	*In vitro* (human sarcoma cell lines)	To investigate the effects of propranolol and carvedilol on the radiosensitivity and viability of sarcoma cells	Propranolol and carvedilol	Primary sarcoma	Both agents induced a concentration-dependent improvement in the viability of sarcoma cells (four lines), with carvedilol being the most potent agent. Chronic low-dose exposure to carvedilol may enhance osteosarcoma cell death
Wrobel et al., 2020 [21]	*In vivo* (Human melanoma biopsies and melanoma cell models)	To evaluate the effect of exposure to various types of β-blockers on the local immune pattern and melanoma progression of the tissues of 20 patients	Cardioselective, wide-spectrum β-blockers	Melanoma	Switch of the local immune system to cytotoxic action when wide-spectrum β-blockers are used
Cecilio et al., 2020 [22]	*In vivo* (4-nitroquinoline-1-oxide -induced oral carcinogenesis rat model)	To study whether treatment with propranolol inhibits chemically induced oral carcinogenesis in rats	Propranolol	Oral tumour	Use of propranolol reduces chemically induced oral carcinogenesis and tumour progression in rats
Lu et al., 2021 [23]	*In vivo* (UVB-induced murine cutaneous squamous cell carcinoma model)	To study the role of β2-adrenergic receptors and selective antagonists on the prevention of UVB-induced squamous cell tumourigenesis	Selective β2-receptor antagonists	Squamous cell skin cancer	Suppression of the action of beta2-receptors by these agents reduces the number and burden of tumours and incidence of malignant squamous skin lesions from UVB
Porcelli et al., 2020 [24]	*In vitro* (patient-derived liposarcoma and leiomyosarcoma cells)	To determine the expression of β-adrenergic receptors on patient-derived liposarcoma and leiomyosarcoma cells and estimate the action of propranolol and its combination with docetaxel	Propranolol	Liposarcoma/leiomyosarcoma	Propranolol synergizes with chemotherapy agents to inhibit sarcoma cell growth
Solerno et al., 2022 [25]	*In vivo* and *in vitro* (Osteosarcoma cell lines and mouse xenograft model)	To evaluate the *in vitro/in vivo* activity of propranolol in osteosarcoma, as a monotherapy or in combination with metronomic chemotherapy	Propranolol	Osteosarcoma	Exposure of osteosarcoma cells to propranolol in combination with cisplatin led to reduced cell growth, mitosis, and G0/G1 cell-cycle arrest, and impaired 2D/3D tumour colony formation and growth
Lin et al., 2023 [1]	*In vivo* and *in vitro* (colorectal cancer cell lines and tumour-bearing mice)	To evaluate the antitumour activity of propranolol when combined with the first-line treatment, irinotecan	Propranolol	Colorectal cancer	Propranolol reverses T-cell exhaustion and enhances the regression of colorectal tumour growth when combined with irinotecan
Fonte et al., 2023 [26]	*In vitro* cervical cancer cell cultures and patient-derived organoids	To evaluate the effect of propranolol on trabectedin efficacy and tumour immunogenicity	Propranolol	Cervical cancer	The combination with propranolol increased the effectiveness of trabectedin
Terzi and Urhan-Kucuk, 2023 [29]	*In vitro* (A549 lung adenocarcinoma cells and BEAS2B normal lung epithelial cells)	To evaluate the anti-proliferative *in vitro* role of propranolol in human lung cancer cells and nontumoural lung cells	Propranolol	Lung tumour	Propranolol reduces cell viability by caspase-dependent and independent apoptosis in lung tumour cells. In nontumoural cells, it has minor cytotoxicity
Yang et al., 2024 [52]	*In vivo* and *in vitro* (melanoma cell lines and murine melanoma models)	To study the anti-oncogenic role and mechanism of action of nebivolol	Nebivolol	Melanoma	Nebivolol induces apoptosis, suppresses proliferation and metastatic effects through downregulation of the expression of MMP2 and enhancement of the metastasis-blocking protein Tissue Inhibitor of Metalloproteinases-2
Dang et al., 2025 [30]	*In vivo* (lung cancer mouse model)	To screen the stimulator of interferon genes (STING) and the role of carvedilol as an effective STING activator	Carvedilol	Lung cancer	Carvedilol acts as a STING activator, promoting the induction of the type I IFN response. Synergistic effect with etoposide
Huang et al., 2025 [31]	*In vitro* and *in vivo* (DLBCL cell lines and lymphoma mouse models)	To study the role of Epstein–Barr virus in diffuse large B-cell lymphoma	ICI118551 (β2 antagonist), SR59230A (β3 antagonist)	Diffuse large B-cell lymphoma (DLBCL)	Diffuse large B-cell lymphoma (DLBCL) patients demonstrated enhanced sympathetic nerve innervation and expression of β2-adrenergic receptors. β-Blockers contribute to improved cancer-specific mortality
Shahid et al., 2025 [53]	*In vivo* (β1/β2-adrenergic receptor knockout mice exposed to UV radiation)	To study the role of β2-adrenergic receptors regarding the effects of UV radiation on skin carcinogenesis	No pharmacological agent (knockout mice for β1 and β2 adrenergic receptors)	Skin carcinogenesis	UV radiation elicits local production of catecholamine, activates β-adrenergic receptors, whereas β-blockers inhibit its action on skin inflammation and injury
Zhu et al., 2026 [54]	*In vitro* and *in vivo* (canine mammary tumour cell lines and tumour-bearing animal models)	To investigate whether propranolol enhances the anti-oncogenic role of Newcastle disease virus on canine mammary tumour cells	Propranolol	Canine mammary tumour	Propranolol enhances the replication and oncolytic activity of Newcastle disease virus by suppressing the antiviral response. Results from *in vivo* studies did not demonstrate additional benefits regarding survival or tumour growth, but highlight the increase in viral load in tumour tissues

### 3.2. Clinical Studies

Several studies have reported improved cancer control in patients treated with β-blockers across various tumour types and treatment phases, either alone or combined with other agents, with or without tumour resection [55,56,57,58,59,60] (Table 2). Spera et al. presented results from the ROSE/TRIO-012 study on breast cancer. They revealed better outcomes in patients under β-blockers, especially those with triple-negative cancer, who were exposed to these agents after enrolment in the study [58]. These findings seem to be valuable, considering that in patients with triple-negative breast cancer, chemotherapy is the only treatment option.

Results from the EORTC 1325/KEYNOTE-054 phase III trial highlight both the predictive value and prognostic significance of β-blockers in patients with resected stage III melanoma [62]. β-Blockers contribute to the efficacy of pembrolizumab but do not affect the recurrence-free survival. Moreover, the postoperative repurposed use of propranolol and etodolac, a COX-2 inhibitor, was studied in the phase II PROSPER trial, which recruited patients with partial pancreatoduodenectomy treated for pancreatic cancer. Although this trial was not completed due to low patient recruitment, several encouraging findings—including improved distal recurrence outcomes, overall survival, and disease-free survival in patients treated with β-blockers in combination with etodolac—warrant further investigation [60]. However, findings from other trials failed to prove benefits from use in patients with platinum-sensitive ovarian cancer [55] or malignant melanoma when used after diagnosis [56].

An overview of clinical studies evaluating the use of β-blockers in cancer patients is presented in Table 2.

### 3.3. Meta-Analyses, Clinical and Retrospective Studies

According to our literature review, several clinical studies [55,56,57,58,59,60,61,62] (Table 2), nine review articles and meta-analyses, and several retrospective studies (Table 3 and Table 4) [48,63,64,65,66,67,68,69,70,71,72,73,74,75,76,77,78,79,80,81] focus on the role of β-blockers in long-term survival or disease-free survival in breast, prostate, or urinary cancer [51,73,74,75,76] (Table 3). According to data from an observational study by Udumyan et al., non-selective β-blockers are associated with better survival rates in patients with urothelial tumours [70]. Preoperative use of β-blockers in emergency colonic tumour surgery may enhance overall or progression-free survival or inhibit tumour progression when combined with other agents, including irinotecan [1,69]. Similarly, their use is associated with improved survival in patients with pancreatic adenocarcinoma, with a corresponding improvement in overall survival [68,71]. A systematic review and meta-analysis showed strong evidence that β-blockers improve long-term survival in patients with hepatocellular carcinoma [77]. However, a meta-analysis of 36 studies and 319,006 patients found no evidence of association between β-blocker use and overall survival, all-cause mortality, disease-free survival, progression-free survival, and recurrence-free survival. When analysis was performed for specific cancer types, a predominantly longer overall survival was found in ovarian cancer, pancreatic cancer, and melanoma, but not in lung, breast, colorectal, or mixed cancer types [74]. Similarly, β-blocker use appears to be associated with relapse-free survival—but not overall survival—in patients with breast cancer, including those with triple-negative disease [65], and no association was observed in non-small-cell lung cancer [66]. In contrast, another study found no association between β-blocker use and improved progression-free or overall survival in pancreatic ductal adenocarcinoma [71]. In another meta-analysis of 27 studies, β-blocker use did not affect cancer recurrence but was associated with improved disease-free survival and overall survival in melanoma, endometrial cancer, and ovarian cancer, but reduced overall survival in head and neck, lung, and prostate cancer [75]. The latter contrasts with another meta-analysis of four studies, including 16,825 patients, showing that β-blocker use was associated with reduced prostate cancer-specific mortality [51]. In a most recent meta-analysis, 79 studies including 492,381 patients showed that use of β-blockers was associated with improved progression-free survival, with significance maintained after excluding high-bias studies. However, no significant associations were observed for overall survival or cancer-specific survival. The most frequently studied cancers were breast, ovarian, and colorectal [76].

Emerging evidence also suggests that β-blockers may influence the response of tumour cells to radiotherapy. As summarised in Table 4, preclinical studies have demonstrated that propranolol and carvedilol can modulate tumour cell survival following radiation exposure and may enhance radiosensitivity in certain tumour models. Proposed mechanisms include attenuation of stress-induced β-adrenergic signalling, modulation of inflammatory responses, inhibition of pro-survival pathways, and effects on the tumour microenvironment [48,64].

**Table 3:** Meta-analyses on the clinical effect of β-blockers on tumour progression and survival.

Study	Type of Article	Purpose of the Study	Results
Lu et al., 2015 [51]	Review and meta-analysis	To assess the efficacy of β-blockers on mortality in patients with prostate cancer	Improved cancer-specific mortality among prostate cancer patients on β-blockers. No effect of β-blockers on all-cause mortality
Weberpals et al., 2016 [73]	Systematic review/meta-analysis	To review evidence on the association between pre- and post-diagnostic β-blocker exposure and cancer survival	Results did not support a role of β-blockers in tumour prognosis, due to methodological limitations, including immortal time
Na et al., 2018 [74]	Meta-analysis	To evaluate the association between the administration of β-blockers and cancer prognosis	Findings from 36 studies investigating the relationship between β-blocker use and cancer prognosis showed no association with overall survival, all-cause mortality, or disease-free survival, while a significant correlation was observed with improved long-term cancer-specific survival
Yap et al., 2018 [75]	Meta-analysis	To study the association between the use of β-blockers, cancer recurrence, overall survival, and disease-free survival	No consistent link was observed between β-blocker use and survival or recurrence across all cancer types. Improved outcomes were noted in certain cancers, such as melanoma and ovarian cancer, whereas poorer outcomes were seen in others, including lung, head and neck, prostate, and endometrial cancers
Sharma et al., 2025 [76]	Systematic review and meta-analysis	To analyse survival outcomes from the use of β-blockers in patients with cancer	Statistically significant findings indicated an association between β-blocker use and progression-free survival across various cancer types, accounting for immortal time bias. A trend toward an association was observed for cancer-specific survival, but not for overall survival
Eraslan et al., 2026 [78]	Review article and meta-analysis	To evaluate the role of β-blockers on survival outcomes in cases with all stages of solid cancers	Use of β-blockers is associated with improved overall survival and progression-free survival in patients with solid tumours
Crispim et al., 2026 [79]	Review article	To study the regulatory mechanism of the nervous system on pancreatic ductal adenocarcinoma	Beta-blockade associated with reduced peri-neural invasion and improved survival rates in cases of pancreatic ductal adenocarcinoma resection
Giri et al., 2026 [80]	Meta-analysis	To study the relationship between β-blocker usage and pancreatic cancer prevention and prognosis	Protective role of β-blockers on the development of pancreatic cancer. No survival effect when used before diagnosis; benefit demonstrated when usage continues after diagnosis
Ni et al., 2026 [81]	Review article	To reveal the role of the nervous system as a regulator of breast cancer initiation, progression, and metastatic growth	Intervention of the β-adrenergic pathway enhances antitumour immune response, suggesting the usage of a combination of neuroregulatory treatment with immunotherapy as a future promising option

**Table 4:** Retrospective studies on the effect of β-blockers on patients’ prognosis and survival.

Study	Purpose of the Study	Tumour Studied	Results
Shah et al., 2011 [63]	To evaluate overall survival outcomes from the use of β-blockers in patients with cancer compared with those under other antihypertensive agents	Solid tumours (breast, lung, stomach, oesophagus, colorectal, renal, prostate, ovary)	Findings not consistent with better overall survival among patients on β-blockers
Melhem-Bertrandt et al., 2013 [65]	To study the role of β-blocker intake on pathological complete response and survival in patients with breast cancer	Breast cancer (invasive)	No difference in pathological complete response rate between the group with β-blocker intake and the group without. Better relapse-free survival in patients on β-blockers. Highly significant association in patients with triple-negative breast cancer, no association in oestrogen receptor-positive breast cancer
Cata and Villareal 2014. [66]	To evaluate results from the perioperative use of β-blockers in cases of lung resection for primary non-small-cell lung cancer	Non-small-cell lung cancer	Use of β-blockers within 30 days of non-small-cell lung tumour resection is not associated with better outcomes regarding recurrence-free and overall survival
Hwa et al., 2017 [67]	To study the impact of β-blockers on overall survival and disease-specific survival of patients with multiple myeloma	Multiple myeloma	Use of β-blockers is associated with improved overall survival, disease-specific survival, and an independent prognostic benefit
Udumyan et al., 2017 [68]	To study the association between the use of β-blockers and cancer-specific mortality rate in pancreatic adenocarcinoma	Pancreatic adenocarcinoma	Use of β-blockers is associated with improved survival, particularly among older patients
Ahl et al., 2018 [69]	To study whether preoperative β-blockers reduced mortality after emergency colonic cancer surgery	Colonic cancer	Preoperative β-blockers in patients undergoing emergency colonic cancer surgery may reduce postoperative mortality/improve surgical outcomes
Udumyan et al., 2022 [70]	To study the association between β-blockers and bladder cancer-specific mortality	Urothelial cancer	The use of non-selective β-blockers is associated with a lower disease-specific mortality rate. Use of selective ones is associated with a slightly better overall mortality rate, not in stage-stratified analyses
Le Bozec et al., 2023 [71]	To assess the role of β-blockers on survival outcomes in patients with pancreatic ductal adenocarcinoma.	Pancreatic ductal adenocarcinoma	No association between β-blocker use and overall or progression-free survival in advanced cases of tumours
Liang et al., 2025 [48]	To present the results of a retrospective study on the impact of β-blockers in patients with non-small-cell lung cancer (NSCLC) following treatment with radiotherapy	NSCLC	Improved overall survival and progression-free survival in patients using β-blockers after radiotherapy, considering the protective role of β-blockers on radiotoxicity from radiotherapy. The benefit in the broader population has not been proven
Chen et al., 2025 [72]	To study the effect of β-blockers on 28-day and 180-day mortality of patients in the Intensive Care Unit treated with this agent, and their impact on tumour-related biomarkers	Malignant tumours	Beta-blockers in patients with cardiovascular comorbidities seem to significantly reduce mortality rates and increase survival rates after 28 and 180 days. They do not appear to affect oncological biomarkers significantly
Crnovrsanin et al., 2025 [64]	To evaluate the effect of β-blockers on gastric tumour outcomes and response to chemotherapy	Gastric cancer	Patients with diffuse gastric cancer under β-blockers presented better overall survival and recurrence-free survival, compared with non-users, with no impact on the response to chemotherapy

## 4. Discussion

### 4.1. β-Blockers and Inhibition of Tumour Growth

**Figure 1:**
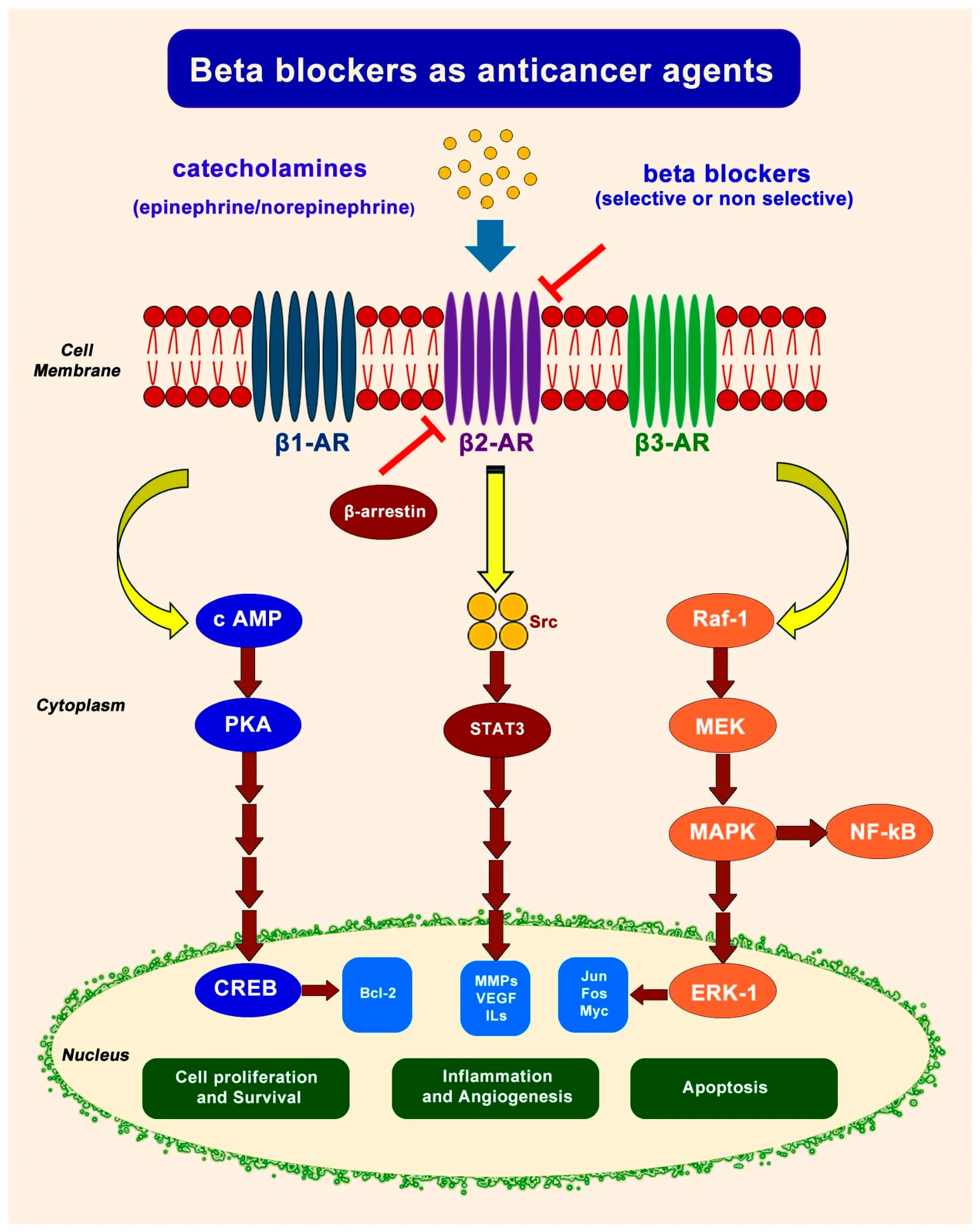
Beta-adrenergic pathway in cancer cellular microenvironment. Catecholamines bind to β-adrenergic receptors, leading to conversion of ATP in cAMP that triggers the PKA downstream signalling, upregulation of transcription factors such as Bcl-2 and induction of cell proliferation [7,55,81,82]. The induction of β-arrestin leads to transient desensitisation of β-receptor signalling, resulting in the activation of mitogenic signalling and the production of transcription factors and pro-inflammatory cytokines, such as STAT3 and MMPs and VEGF respectively, modulating inflammation and angiogenesis [13]. The Raf-1/MEK/MAPK/NFκΒ pathway upregulates the expression of transcription factors involved in cellular survival and apoptotic process [10,13] (cAMP: cyclic adenosine monophosphate, ERK: extracellular signal-regulated kinase, MAPK: mitogen-activated protein kinase, MMPs: matrix metalloproteinases, NFκB: Nuclear Factor kappa Beta).

Recent evidence highlights a central role of β-adrenergic signalling in both tumour and stromal cells in promoting cancer progression through mechanisms such as tumour cell proliferation, angiogenesis, extracellular matrix remodelling, invasion, and immunosuppression [82,83]. This has led to the hypothesis that β-blockers may serve as promising adjuvant agents in cancer treatment strategies. Indeed, β-blockade suppresses VEGF-mediated angiogenesis, thereby limiting the delivery of nutrients and oxygen to tumours [84]. In addition, inhibition of β-adrenergic signalling reduces activation of the cAMP–PKA and MAPK/ERK pathways, ultimately impairing tumour cell proliferation and survival. Furthermore, β-blockers tend to restrict MMPs and epithelial-to-mesenchymal transition, reducing metastatic potential and the ability of tumour cells to invade surrounding tissues [11] (Figure 1). β-Adrenergic signalling has also been linked to activation of NFκΒ and signal transducer and activator of transcription 3 (STAT3), both of which contribute to chronic inflammation, tumour growth, and angiogenesis [13]. Inhibition of β-adrenergic signalling has also been associated with reduced recruitment of immunosuppressive cells, restoration of cytotoxic T-lymphocyte and natural killer-cell activity, and attenuation of protumour inflammatory responses. These immunomodulatory effects may partly explain the growing interest in combining β-blockers with immune checkpoint inhibitors and other immunotherapeutic strategies [12,15]. Collectively, β-blockade may exert antitumour effects not only through direct inhibition of tumour cell proliferation but also through broader modulation of the tumour microenvironment [10,11,13].

Clinical studies tend to confirm the preclinical results. Observational studies on breast cancer have demonstrated that usage of non-selective β-blockers is associated with improved relapse-free survival and lower recurrence rates [65]. Similarly, β-blocker therapy has been associated with slower disease progression and a reduced incidence of metastasis in melanoma [85]. Interestingly, the magnitude of benefit appears most pronounced with non-selective β-blockers, supporting the hypothesis that inhibition of β2-adrenergic signalling represents a key mechanism underlying their anticancer activity [12,16].

The perioperative period represents a particularly vulnerable period in cancer progression, and surgical stress induces an acute catecholamine peak, which may facilitate residual tumour cell survival and metastatic expansion. Perioperative administration of β-blockers has been shown to eliminate these effects, according to the results from various clinical studies, suggesting a potential role in reducing post-surgical recurrence [74,75]. This underscores the importance of timing and clinical context when using β-blockers as adjunctive therapy.

Despite promising evidence, several challenges remain before β-blockers can be routinely incorporated into oncological practice. Optimal dosing regimens, treatment duration, and patient selection criteria have not yet been established. Moreover, the current evidence base is dominated by preclinical investigations and retrospective observational studies, whereas prospective randomised clinical trials remain limited. Consequently, many of the reported associations should still be considered hypothesis-generating rather than definitive evidence of clinical benefit. Future studies should focus on identifying predictive biomarkers, including tumour-specific β-adrenergic receptor expression profiles, and evaluating biomarker-guided approaches to patient selection. A better understanding of pharmacogenetic variability, receptor subtype expression, and interactions with chemotherapy, immunotherapy, radiotherapy, and targeted therapies may facilitate the development of more personalised and effective β-blocker-based treatment strategies in oncology.

### 4.2. β-Blockers as an Adjuvant Therapy

The use of β-blockers as adjuvant therapy in oncology is increasingly recognised, particularly in combination with chemotherapy and immunotherapy. Clinical and preclinical evidence demonstrates that β-blockers can enhance chemosensitivity and reduce metastatic potential. Results from breast and ovarian cancer models reveal that propranolol combined with paclitaxel, doxorubicin, or cisplatin resulted in greater tumour suppression compared to chemotherapy alone [8,11,86]. β-Blockers counteract stress-induced chemoresistance, thereby potentiating cytotoxic therapies [84]. Moreover, they modulate the tumour immune microenvironment, enhancing the efficacy of immunotherapy. By restoring cytotoxic T- and NK-cell activity, which are often suppressed under chronic stress, β-blockers may enhance host antitumour immune responses [8,87]. Preclinical melanoma and breast cancer studies indicate improved responses to checkpoint inhibitors when β-blockers mitigate stress-mediated immunosuppression, with perioperative administration reducing the risk of metastasis [83]. Safety, patient selection, and optimal dosing and timing, particularly perioperatively or in combination with systemic therapies, remain critical considerations.

### 4.3. Selective Versus Non-Selective β-Blockers

The pharmacological properties of individual β-blockers may significantly influence their anticancer activity. Although all β-blockers inhibit adrenergic signalling, important differences exist in receptor selectivity, downstream signalling effects, tissue distribution, and pleiotropic properties. Non-selective β-blockers, such as propranolol and nadolol, inhibit both β1- and β2-adrenergic receptors, whereas selective agents, including atenolol, metoprolol, and bisoprolol, primarily target β1 receptors. Increasing evidence suggests that β2-adrenergic signalling plays a predominant role in tumour progression by promoting cell proliferation, angiogenesis, epithelial–mesenchymal transition, invasion, metastasis, and immune evasion. Consequently, blockade of β2-adrenergic receptors may be particularly important for achieving antitumour effects [13,83].

Several preclinical studies have demonstrated greater anticancer activity with non-selective β-blockers than with β1-selective agents. Propranolol has consistently shown inhibitory effects on tumour growth, angiogenesis, and metastatic dissemination across multiple experimental models, whereas selective β1-blockers have generally produced less pronounced effects [23,24,25]. These findings support the hypothesis that simultaneous inhibition of both β1- and β2-adrenergic receptors may provide broader suppression of protumour adrenergic signalling pathways [13]. In addition to receptor selectivity, certain β-blockers possess pleiotropic properties that may further influence tumour biology. Specifically, carvedilol exhibits antioxidant activity and α1-adrenergic receptor antagonism, whereas nebivolol enhances nitric oxide bioavailability and endothelial function [30,38,52]. These properties may contribute to modulation of oxidative stress and the tumour microenvironment, although their clinical relevance in oncology remains incompletely understood. Despite the apparent advantages of non-selective β-blockers, the optimal agent may vary depending on tumour type, receptor expression profile, patient characteristics, and concomitant therapies. Future studies should therefore focus on identifying tumour-specific patterns of adrenergic receptor expression and determining whether receptor selectivity can serve as a predictive factor for therapeutic response. Such an approach may facilitate the development of more personalised β-blocker-based strategies within precision oncology.

### 4.4. β-Blockers and Patient Outcome

Although β-blocker use appears to improve overall survival across various tumour types, the evidence remains inconsistent, at least partly due to the retrospective design of these studies and the inclusion of populations who are already treated with β-blockers, mainly for cardiovascular diseases [88]. The existing studies are highly heterogeneous and mainly observational, while there is a shortage of specific randomised clinical trials. Another source of complexity is the differences in the concentrations of β1-, β2-, and β3-adrenergic receptors, and thus in sensitivity to catecholamines, between tumour types, which could explain variations in the clinical benefit of β-blockers [76]. Moreover, no association was found between β-blocker use and overall survival in patients with solid tumours compared with those receiving other antihypertensive agents [63], raising questions about the efficacy of β-adrenergic receptor blockade independent of its cardiovascular effects. 

The apparent beneficial effect of β-blockers on cancer mortality tends to be attenuated when studies [39,72] affected by immortal time bias are excluded, despite some of these studies showing a substantial reduction in mortality [73]. Immortal time bias arises from periods during which patients are “immortal” to the outcome, and its impact depends on both the duration of immortal time and the prognosis associated with each tumour type. When analyses properly consider immortal time and other methodological factors, the evidence does not consistently support a protective role for β-blockers in tumour prognosis [89].

Moreover, findings from other studies on the efficacy and long-term outcomes of beta-blockers in non-perioperative and perioperative settings are inconsistent relative to tumour recurrence and survival. Improved outcomes appear to be limited to specific tumour subtypes, while other tumours may show no benefit or even adverse effects, reflecting the heterogeneity of β-blocker action [75]. This variability is compounded by the presence of different β-adrenergic receptor subtypes (β1, β2, and β3) on cancer and immune cells, as well as differences in the selectivity of the β-blockers employed [90].

### 4.5. Future Perspectives

Despite growing interest in the repositioning of β-blockers as anticancer agents, several important questions remain unanswered. Current evidence suggests that the therapeutic potential of β-blockers may vary considerably across tumour types. The strongest support currently exists for breast cancer, melanoma and angiosarcoma, where preclinical studies and retrospective clinical investigations have consistently reported reductions in tumour progression, recurrence, or metastasis [11,16,85]. However, predicated on the presented data, the impact of β-blockers on the management of ovarian cancer is controversial [7,55,83]. In contrast, findings in lung, colorectal, prostate, and other malignancies remain less consistent, highlighting the need for further validation in prospective clinical trials.

One of the major challenges in the clinical development of β-blockers in oncology is the identification of patient populations most likely to benefit from treatment. Tumour-specific expression of β-adrenergic receptor subtypes, particularly β2-adrenergic receptors, may influence therapeutic responsiveness and could serve as a predictive biomarker for treatment selection. Future studies should investigate biomarker-guided approaches that integrate receptor expression profiles, tumour molecular characteristics, and features of the tumour microenvironment to facilitate more personalised treatment strategies [13,83].

Pharmacogenetic variability may represent another important determinant of treatment efficacy and safety. Genetic polymorphisms affecting β-adrenergic receptors or enzymes involved in β-blocker metabolism, such as CYP2D6, may influence drug response and contribute to interindividual variability in therapeutic outcomes. Variability in drug transporters and metabolising enzymes has been shown to influence β-blocker pharmacokinetics, highlighting the potential importance of personalised dosing strategies [91]. Although pharmacogenetic testing has not yet been systematically evaluated in the context of β-blocker repositioning for cancer therapy, recent advances in precision medicine suggest that such approaches may improve patient selection and treatment optimisation in future clinical studies.

The ability of β-blockers to modulate angiogenesis, immune responses, stress-related signalling, and tumour cell survival pathways provides a strong rationale for combination strategies. Emerging evidence suggests that β-blockers may enhance the efficacy of chemotherapy, radiotherapy, targeted therapies, and immune checkpoint inhibitors by reducing protumour adrenergic signalling and modifying the tumour microenvironment [77]. Recent real-world data in advanced lung cancer demonstrated improved outcomes among patients receiving β-blockers during immune checkpoint inhibitor therapy [12]. In particular, the growing interest in combining β-blockers with immunotherapy warrants further investigation, as preclinical and early clinical studies indicate that β-adrenergic blockade may help restore antitumour immune responses and improve treatment outcomes. Moreover, advances in cancer immunology and epigenetics have highlighted the potential of novel epigenetic immunotherapies to enhance antitumour immunity, particularly when combined with established treatment modalities [92]. The broad therapeutic application of β-blockers across different clinical settings underscores the potential importance of pharmacogenetic variability and biomarker-guided treatment strategies. Although these approaches have been explored in other fields of medicine, their role in oncology remains largely unexplored and warrants further investigation [93].

Future research should focus on well-designed prospective randomised clinical trials to establish the optimal β-blocker agent, dose, treatment duration, and timing of administration. A better understanding of tumour biology, receptor subtype expression, pharmacogenetic variability, and interactions with existing anticancer therapies will be essential to translate promising preclinical findings into effective clinical applications. Such efforts may ultimately define the role of β-blockers as cost-effective and widely available agents within precision oncology frameworks [13,77].

### 4.6. Strengths and Limitations

To our knowledge, this is the most up-to-date review, incorporating the latest preclinical and clinical data, systematic reviews, and meta-analyses on the repurposed use of β-blockers as an adjuvant treatment strategy across multiple tumour types. The included studies address key clinical outcomes regarding overall survival, mortality, recurrence, and metastasis. These studies primarily focus on specific β-blockers and their underlying mechanisms of action, drawing on evidence from experimental investigations. The synergistic effects of β-blockers across multiple oncologic therapeutic targets are examined, with supporting evidence derived from both *in vitro* and *in vivo* studies spanning a broad range of malignancies. However, heterogeneity among study populations, study designs, methodologies, cancer stages, and tumour types represents an inherent limitation of this review. As a narrative review, this study aims to highlight the most recent advances in the repurposed use of β-adrenergic antagonists, offering a comprehensive overview of their potential integration into contemporary oncologic practice.

## 5. Conclusions

β-Blockers show promising potential as adjuvant cancer therapies by inhibiting β-adrenergic signalling, which contributes to tumour growth, metastasis, angiogenesis, and immune evasion. Clinical and preclinical studies suggest they may enhance the efficacy of chemotherapy, immunotherapy, and perioperative interventions in selected cancers, including breast, melanoma, pancreatic, and in some cases, lung tumours. Benefits are tumour-specific, and some trials, such as in ovarian cancer, have shown limited efficacy, underscoring the need for further investigation. Several ongoing clinical trials are now evaluating β-blockers in combination with standard therapies across different tumour types, aiming to identify optimal dosing, timing, and responsive patient subgroups [55,57,59,60,61,62]. Future well-designed clinical and mechanistic studies are essential to define patient populations, clarify antitumour mechanisms, and establish β-blockers as cost-effective, repurposed therapies in oncology.

## Figures and Tables

**Table 2:** Data from clinical studies on the repurposed use of β-blockers.

Study	Purpose of the Study	Β-Blocker Used	Tumour Studied	Results
Heitz et al., 2013 [55]	To evaluate the impact of β-blockers in cases of recurrent platinum-sensitive ovarian cancer	Metoprolol, bisoprolol, atenolol, talinolol, betaxolol, nebivolol, penbutolol, sotalol, acebutolol, propranolol	Ovarian cancer	Treatment with β-blockers was not associated with better outcomes in patients with platinum-sensitive ovarian cancer
Mccourt et al., 2014 [56]	To estimate post-diagnostic use of β-blockers and mortality in patients with malignant melanoma	Atenolol, bisoprolol, metoprolol, propranolol, sotalol	Melanoma	No association with all-cause survival and melanoma-specific survival; only atenolol presented an increased odds ratio of all-cause mortality, but not melanoma-specific mortality
Labreze et al., 2015 [57]	To evaluate the efficacy and safety of the use of propranolol in cases of infantile haemangioma	Propranolol	Infantile haemangioma	Effective use of propranolol in infantile haemangioma with a favourable profile of benefit to risk
Spera et al., 2017 [58]	To study the role of β-blockers on overall survival in localised breast cancer	Bisoprolol, metoprolol, atenolol, propranolol	Breast cancer	Better outcomes in progression-free survival in cases of advanced breast cancer, especially when β-blockers are administered after enrolment in the study in triple-negative cases
Haldar et al., 2020 [61]	To study perioperative pathways of blockade of prostaglandins and catecholamines in patients with colorectal cancer	Propranolol	Colorectal cancer	A combination of propranolol and a COX2 inhibitor achieved favourable results regarding molecular biomarkers related to tumour growth and metastasis
Kennedy et al., 2022 [62]	To evaluate the predictive and prognostic value of β-blockers in pembrolizumab versus placebo in a resected high-risk stage III melanoma phase III trial	β1-selective agents (acebutolol, atenolol, betaxolol, bisoprolol, celiprolol, esmolol, metoprolol, nebivolol)/non-selective agents (carteolol, carvedilol, labetatol, levobunolol, metipranolol, nadolol, oxprenolol, penbutolol, pindolol, practolol, propranolol, sotalol, timolol)	Melanoma	No benefit from the use of β-blockers in recurrence-free survival in patients with stage III high-risk resected melanoma. Use of these agents increases the efficacy of pembrolizumab
Huettner et al., 2025 [60]	To study the safety endpoints of using a COX-2 inhibitor and propranolol in the perioperative pancreatic cancer (phase II randomised controlled PROSPER trial)	Propranolol	Pancreatic cancer	Although this study was not concluded due to low recruitment, some interesting results, including longer disease-free and overall survival, and fewer distant metastases compared with the placebo group, merit further study

## Data Availability

No new data were created or analysed in this study. Data sharing is not applicable to this article.
